# Surface Modification of Li(Ni_0.6_Co_0.2_Mn_0.2_)O_2_ Cathode Materials by Nano-Al_2_O_3_ to Improve Electrochemical Performance in Lithium-Ion Batteries

**DOI:** 10.3390/ma10111273

**Published:** 2017-11-06

**Authors:** Kwang Soo Yoo, Yeon Hui Kang, Kyoung Ran Im, Chang-Sam Kim

**Affiliations:** 1 Department of Materials Science and Engineering, University of Seoul, 163, Seoulsiripdae-ro, Dongdaemun-gu, Seoul 02504, Korea; yunhee813@naver.com; 2 Center for Energy Convergence Research, Korea Institute of Science and Technology, 5, Hwarang-ro 14-gil, Seongbuk-gu, Seoul 02792, Korea; 3 G-Materials Co., Ltd., 649, Ori-ro, Gwangmyeong-si, Gyeonggi-do 14303, Korea; krhankyoung@gmail.com

**Keywords:** lithium-ion batteries, Ni-rich cathode materials, nano-Al_2_O_3_ coating, surface modification, electrochemical property

## Abstract

Al_2_O_3_-coated Li(Ni_0.6_Co_0.2_Mn_0.2_)O_2_ cathode materials were prepared by simple surface modification in water media through a sol-gel process with a dispersant. The crystallinity and surface morphology of the samples were characterized through X-ray diffraction analysis and scanning electron microscopy observation. The Li(Ni_0.6_Co_0.2_Mn_0.2_)O_2_ cathode material was of a polycrystalline hexagonal structure and agglomerated with particles of approximately 0.3 to 0.8 μm in diameter. The nanosized Al_2_O_3_ particles of low concentration (0.06–0.12 wt %) were uniformly coated on the surface of Li(Ni_0.6_Co_0.2_Mn_0.2_)O_2_. Measurement of electrochemical properties showed that Li(Ni_0.6_Co_0.2_Mn_0.2_)O_2_ coated with Al_2_O_3_ of 0.08 wt % had a high initial discharge capacity of 206.9 mAh/g at a rate of 0.05 C over 3.0–4.5 V and high capacity retention of 94.5% at 0.5 C after 30 cycles (cf. uncoated sample: 206.1 mAh/g and 90.8%, respectively). The rate capability of this material was also improved, i.e., it showed a high discharge capacity of 166.3 mAh/g after 5 cycles at a rate of 2 C, whereas the uncoated sample showed 155.8 mAh/g under the same experimental conditions.

## 1. Introduction

Current lithium-ion battery technology offers the highest energy density among the rechargeable battery technologies, dominating the market for mobile electronic devices for the past several decades. However, alternative forms of transportation, such as electric and plug-in hybrid electric vehicles, require significant improvements in energy density, safety, durability, cost, etc. The key to the successful development of novel and advanced rechargeable batteries is the materials [1].

Since Sony commercialized the lithium-ion secondary battery (C/LiCoO_2_ cell) in 1991, various cathode (positive electrode) materials, which account for approximately 30% of materials in the lithium-ion battery, have been studied by many investigators. LiCoO_2_ has been the dominating cathode material for commercial lithium-ion batteries owing to its high capacity, stable cycling, and easy production [2]. However, cobalt in LiCoO_2_ is a rare metal, expensive, and toxic, therefore alternative cathode materials such as ternary Li(Ni_1-x-y_Co_x_Al_y_)O_2_ and Li(Ni_1-x-y_Co_x_Mn_y_)O_2_ compounds with layered structures, spinel-structured LiMn_2_O_4_, olivine-structured LiMPO_4_ (M = Fe, Mn, Co, Ni), and orthosilicates Li_2_MSiO_4_ (M = Fe, Mn, Co), have been intensively investigated [1,3,4,5,6,7,8]. Among these candidates, Li(Ni_1-x-y_Co_x_Mn_y_)O_2_ has been considered as a possible replacement for LiCoO_2_ [9,10,11,12].

Nickel-rich (Ni-rich) layered compounds (1 − x − y ≥ 0.5), such as Li(Ni_0.6_Co_0.2_Mn_0.2_)O_2_ are the most promising because high Ni and low Co content contributes to the improvement of specific capacity and the reduction of cost [10]. Recently, Erickson et al. [13] reported that cathodes prepared from Li-rich xLi_2_MnO_3_·(1 − x)Li(Ni_a_Co_b_Mn_c_)O_2_ (a + b + c = 1) demonstrated extremely high discharge capacities. However, as the content of Ni in Li(Ni_1-x-y_Co_x_Mn_y_)O_2_ increases, its thermal, structural, and chemical stabilities decrease [14,15,16,17]. Accordingly, the cathode materials with high capacity and good thermal stability, simultaneously, are necessary. This could be achieved by improving the degradation of the electrochemical properties and thermal stability in lithium-ion batteries caused by the interface reaction between the cathode material and the electrolyte solution. This problem could be solved by coating the surface of the cathode material with a different material. This surface modification technology was introduced for a LiCoO_2_ cathode by coating with metal oxides, such as TiO_2_, Al_2_O_3_, Mg_2_TiO_4_, and NaAlO_2_ [2,18,19,20]; LiNi_0.5_Mn_1.5_O_4_ cathode by ZrO_2_, ZrP_2_O_7_, and AlPO_4_ coating [21,22]; and Li(Ni_0.6_Co_0.2_Mn_0.2_)O_2_ cathode by TiO_2_, Al_2_O_3_, and Li_2_ZrO_3_ coating [23,24,25]. Among the coating processes, wet chemical processes such as sol-gel and precipitation are widely applied, which typically require 0.3–5 wt % of coating material respect to the cathode material and resulted in substantially inhomogeneous coating. Recently ultrasonic-assisted process was introduced to coat Li(Ni_0.6_Co_0.2_Mn_0.2_)O_2_ with nano-Al_2_O_3_; Chen et al. [24] reported that the electrochemical performance of a Li(Ni_0.6_Co_0.2_Mn_0.2_)O_2_ cathode material coated with Al_2_O_3_ of 1.0 wt %showed initial discharge capacity of 197.1 mAh/g over 3.0–4.5 V and capacity retention of 91% after 30 cycles at 1 C. Here γ-Al_2_O_3_ nano-particles (d_50_ ca. 15 nm) and Li(Ni_0.6_Co_0.2_Mn_0.2_)O_2_ powder were dispersed by ultrasonic treatment in ethanol, stirred, and subsequently evaporated the ethanol. Finally the dried sample was heat treated at 500 °C for 6 h. The thickness of the coating layer is about 20–25 nm. Therefore, it is desirable to develop a simple process to provide coating layers, which are substantially thin, discrete, and uniform to provide hindrance of the interfacial reaction of the electrode/electrolyte and little inhibition in the diffusion of Li^+^ ions. 

Herein, we demonstrate the use of simple surface modification in water media to improve the electrochemical properties, especially high capacity and stable cycling, of the Li(Ni_0.6_Co_0.2_Mn_0.2_)O_2_ cathode. The Al_2_O_3_-coated Li(Ni_0.6_Co_0.2_Mn_0.2_)O_2_ cathode materials were prepared by mixing cathode powder with Al_2_O_3_ precursor in water, drying the mixed slurry, and then annealing at various temperatures. The effects of the Al_2_O_3_ coating on the structural and electrochemical properties of the Li(Ni_0.6_Co_0.2_Mn_0.2_)O_2_ cathode material were systematically investigated.

## 2. Experimental Procedure

The Li(Ni_0.6_Co_0.2_Mn_0.2_)O_2_ cathode material (d_10_/d_50_/d_90_ = 7.77/13.38/22.72 μm) was a commercially available powder obtained from Ningbo Jinhe Lithium Battery Material Co., Ltd (Ningbo, China). Aqueous alumina (Al_2_O_3_) sol was prepared with a concentration of 10 wt % by peptizing Boehmite (γ-AlO(OH)) powder in acidic water. This sol is viscous, translucent, and stable for several months. The Li(Ni_0.6_Co_0.2_Mn_0.2_)O_2_ powder was added to a mixture of the alumina sol and an additive of polymeric dispersant based on polyethylene glycol in deionized water. The slurry concentration was approximately 80 wt %. The amount of alumina sol that was used varied from 0.06 to 0.12 wt % as Al_2_O_3_ with respect to the Li(Ni_0.6_Co_0.2_Mn_0.2_)O_2_ powder and the additive 0.04 wt %. The mixture was mixed with a stirrer for 1 h, heated to remove the water while stirring, and then dried in an oven. The dried coated powder was subjected to heat treatment at various temperatures from 400 to 600 °C and holding times varying from 2 h to 10 h. 

The structural properties of the Al_2_O_3_-coated Li(Ni_0.6_Co_0.2_Mn_0.2_)O_2_ cathode materials were characterized by a powder X-ray diffractometer (XRD, Miniflex II, Rikagu, Tokyo, Japan) with a 2θ scan from 10 to 90°, where Cu K_α_ (λ = 1.5414 Å) radiation was used for the X-ray source and the scan rate was 0.5°/min. The surface morphologies of the cathode materials were observed using a scanning electron microscopy (SEM, Nova 200, FEI, Hillsboro, OR, USA) operating at 10 kV and a scanning transmission electron microscopy (STEM, FEI, Talos F200X) operating at 200 kV with an energy dispersive X-ray spectrometer (EDS) for elemental mapping. 

The cathode electrodes were prepared by mixing Al_2_O_3_-coated Li(Ni_0.6_Co_0.2_Mn_0.2_)O_2_ as an active material, carbon black as a conducting material, and polyvinylidene difluoride (PVdF) as a binder with a weight ratio of 90:5:5; In addition, *N*-methyl pyrrolidone (NMP), as a solvent, was added to control the concentration of this slurry. The mixture was applied to aluminum foil and dried at 80 °C for 2 h in a vacuum oven. The dried foil was hot-rolled to improve the adhesion between the active material and aluminum foil, and cut into circular discs of 14 mm in diameter. These discs were placed in a vacuum oven at 80 °C for 24 h to vaporize any NMP that remained in the cathode. 

To measure the electrochemical properties, standard coin cells (2032 type) were assembled inside a dry chamber and lithium foil was used as a counter electrode. The electrolyte was 1 M LiPF_6_ in a solution of ethylene carbonate, ethyl methyl carbonate, and dimethyl carbonate (1:1:1 in volume ratio). The charge and discharge tests were performed at various C rates over the potential range of 3.0 to 4.5 V at room temperature using a galvanostatic cycling system (WBCS-3000, WonAtech, Seoul, Korea). The charge and discharge tests were conducted as one cycle at 0.5 C, 2 cycles at 0.1 C and 0.2 C, 5 cycles at 0.5 C, 1 C, and 2 C, and then 30 cycles at 0.5 C for cycle performance. Thus, in this experiment, the total number of charge and discharge cycles was 53. Tests were carried out with 3 samples, and leave out the off value and take an average of the two.

## 3. Results and Discussion

The strategy attempted here is to produce a very thin and uniform Al_2_O_3_ coating on the Li(Ni_0.6_Co_0.2_Mn_0.2_)O_2_ cathode material by simply modifying a sol-gel process with a dispersant. This was accomplished through the following steps: the preparation of a stabilized coating solution, acid-base adsorption, a sol-gel process to form the thin film on the surface of the Li(Ni_0.6_Co_0.2_Mn_0.2_)O_2_ powder, and a subsequent heat treatment step. Because the aqueous slurry of the Li(Ni_0.6_Co_0.2_Mn_0.2_)O_2_ is basic, an acidic coating solution is preferred. The coating solution should be homogeneous and stable, so that the coating solution can be uniformly adsorbed on the surface of the Li(Ni_0.6_Co_0.2_Mn_0.2_)O_2_ powder. Proper additives are helpful to stabilize the coating solution as well as to disperse the Li(Ni_0.6_Co_0.2_Mn_0.2_)O_2_ powder in water. The adsorbed coating solution yields ultrathin films on the powder via gelling upon drying. These ultrathin films decompose to nano-Al_2_O_3_ upon heat treatment yielding adherent nano-Al_2_O_3_ particulates on the surface of the Li(Ni_0.6_Co_0.2_Mn_0.2_)O_2_ powder. To date, surface modification using wet processes has employed considerably large quantities of the Al_2_O_3_ precursor from 0.3 to 5 wt %. In the present study, we tried with a very low quantity of the Al_2_O_3_ precursor so that the solution coats the surface of the Li(Ni_0.6_Co_0.2_Mn_0.2_)O_2_ powder well and evenly. Moreover, coating of excess Al_2_O_3_ than the appropriate amount that will result in the deterioration of the electrochemical properties can be avoided. 

**Figure 1:**
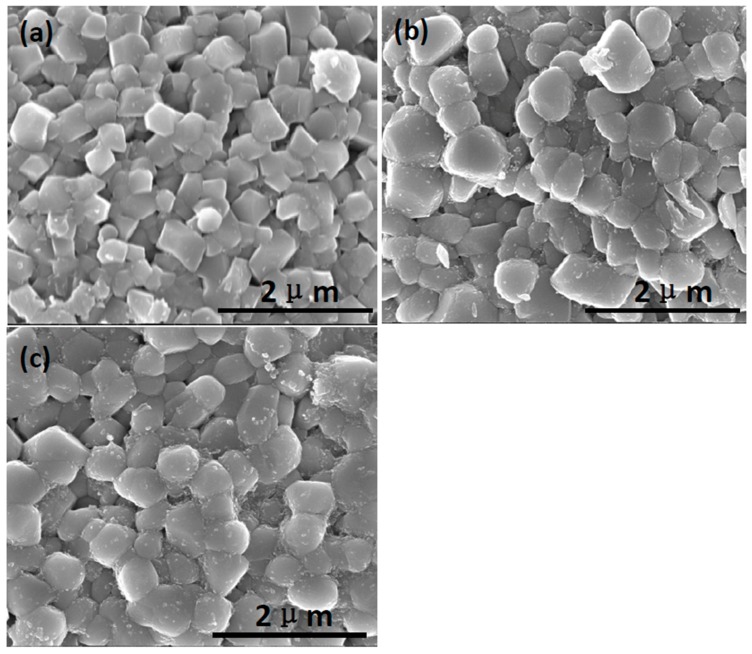
SEM images of the Li(Ni_0.6_Co_0.2_Mn_0.2_)O_2_ powders coated with (**a**) 0.06 wt %; (**b**) 0.08 wt %; and (**c**) 0.12 wt % as Al_2_O_3_, and annealed at 500 °C for 4 h.

**Figure 2:**
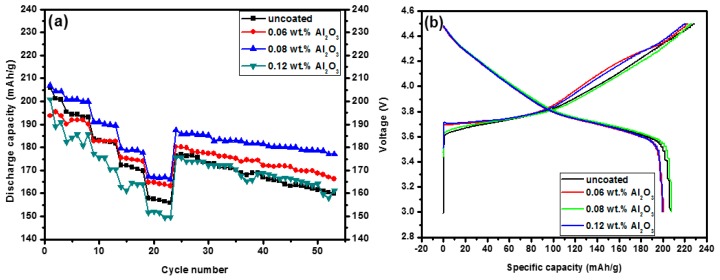

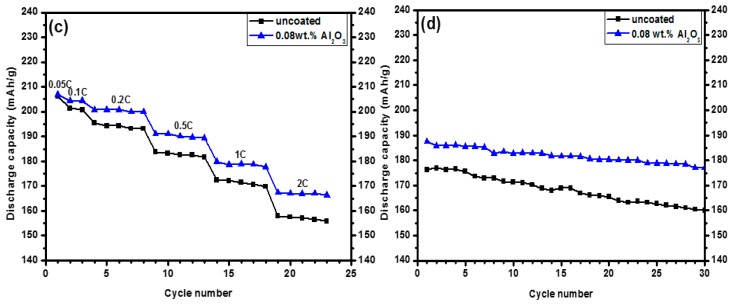
Electrochemical performance of the Li(Ni_0.6_Co_0.2_Mn_0.2_)O_2_ samples uncoated and coated with different amounts of Al_2_O_3_ annealed at 600 °C for 4 h and cycled between 3.0 and 4.5 V; (**a**) Discharge capacity vs. cycle number; (**b**) initial charge and discharge curves; (**c**) rate capability, and (**d**) cycling performance operated at a rate of 0.5 C.

The results obtained by using a low concentration of the coating material are shown in Figure 1. The amount of Al_2_O_3_ in the sol was varied: 0.06 wt % for Figure 1a, 0.08 wt % for Figure 1b, and 0.12 wt % for Figure 1c. As the amount of Al_2_O_3_ increased, the adherent nano-Al_2_O_3_ also increased. The Al_2_O_3_ phase obtained by heat treatment at 500 °C for 4 h was γ-Al_2_O_3_, and this phase was maintained up to 700 °C [26]. The nanoparticles were dispersed uniformly and discretely in all three samples. Because 0.12 wt % of Al_2_O_3_ resulted in too many nanoparticles, 0.08 wt % of Al_2_O_3_ was chosen as a suitable amount for the Li(Ni_0.6_Co_0.2_Mn_0.2_)O_2_ powder. This was also supported by their electrochemical performance for the samples heat treated at 600 °C for 4 h as shown in Figure 2a. Thus, further studies were performed with this composition of 0.08 wt % Al_2_O_3_. 

### 3.1. Structural Properties

**Figure 3:**
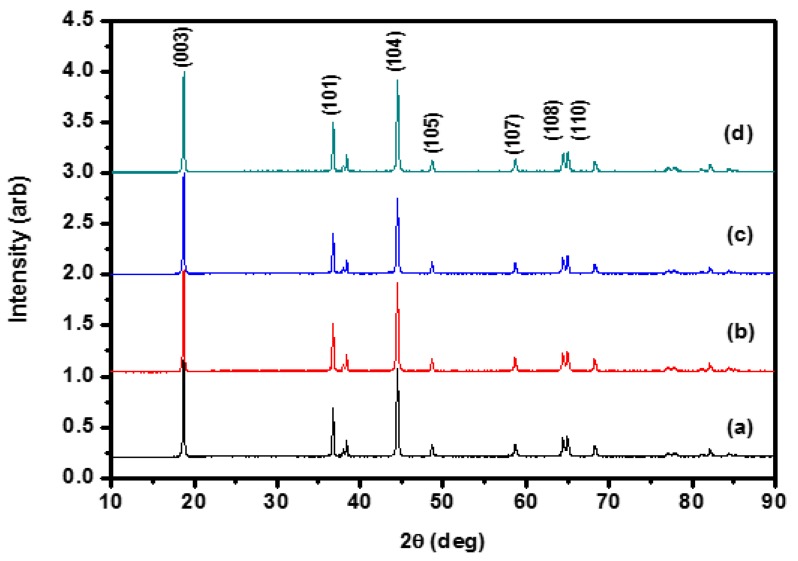
X-ray diffraction patterns of the Li(Ni_0.6_Co_0.2_Mn_0.2_)O_2_ samples uncoated and coated with 0.08 wt % Al_2_O_3_ annealing at 600 °C for 2, 4, and 10 h, respectively. (**a**) Uncoated; (**b**) annealed at 600 °C for 2 h; (**c**) annealed at 600 °C for 4 h; and (**d**) annealed at 600 °C for 10 h.

The XRD analysis of the uncoated and Al_2_O_3_-coated Li(Ni_0.6_Co_0.2_Mn_0.2_)O_2_ samples was performed to determine the effect of the Al_2_O_3_ coatings on the crystal structure of Li(Ni_0.6_Co_0.2_Mn_0.2_)O_2_. Figure 3 shows the XRD patterns of Li(Ni_0.6_Co_0.2_Mn_0.2_)O_2_ uncoated and coated with 0.08 wt % Al_2_O_3_ annealed at 600 °C for 2, 4, and 10 h. As shown in Figure 3a, the uncoated Li(Ni_0.6_Co_0.2_Mn_0.2_)O_2_ sample was a hexagonal α-NaFeO_2_ type structure with a space group of R$\bar{3}$m which agreed well with the JCPDS 87-1564 [27] and many earlier results [9,24,28,29]. There was no significant change in the XRD patterns for the 0.08 wt % Al_2_O_3_ coated Li(Ni_0.6_Co_0.2_Mn_0.2_)O_2_ samples compared to the uncoated Li(Ni_0.6_Co_0.2_Mn_0.2_)O_2_ sample. No impurity peak was observed from the XRD patterns. 

**Figure 4:**
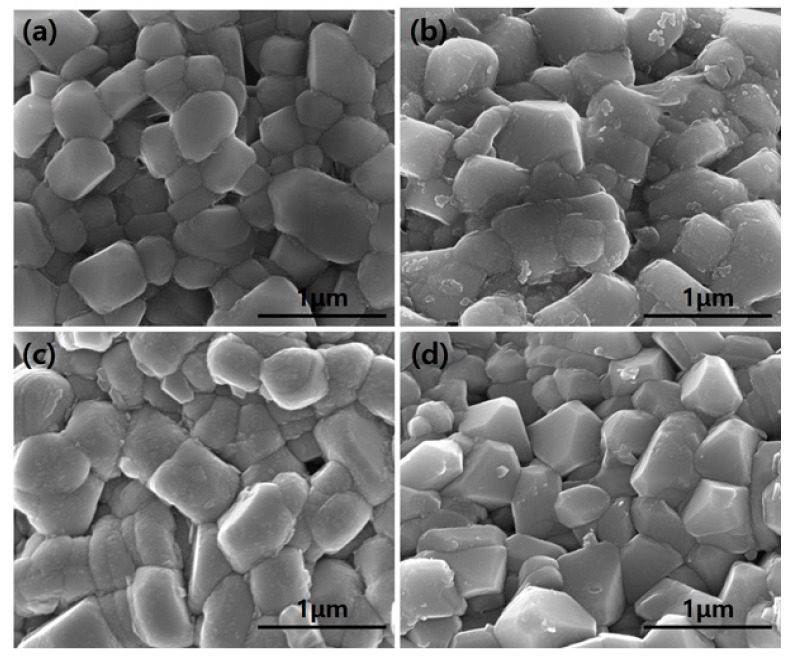
SEM images of the Li(Ni_0.6_Co_0.2_Mn_0.2_)O_2_ samples (**a**) uncoated without heat treatment (dried at 150 °C) and (**b**–**d**) coated with 0.08 wt % Al_2_O_3_ annealing at 600 °C for 2, 4, and 10 h, respectively.

The effects of heat treatment were observed by SEM, and the results are shown in Figure 4. The samples dried at 150 °C (Figure 4a) and the annealed at 600 °C for 2 h (Figure 4b) show uniformly dispersed discrete nano-Al_2_O_3_ coated Li(Ni_0.6_Co_0.2_Mn_0.2_)O_2_ particles while maintaining the morphology of the pristine, but the discrete nano-Al_2_O_3_ particles were mostly disappeared and the sharp-edged morphology was developed after the heat treatment at 600 °C for 4 h (Figure 4c) and for 10 h (Figure 4d). It is noticeable that drying at 150 °C yields discrete adherent Boehmite nano-particles that resulted from the loss of volatiles of the films on the surface of the Li(Ni_0.6_Co_0.2_Mn_0.2_)O_2_ powder. This supports our strategy to produce thin films on the surface via a sol-gel process. Heat treatment at 600 °C transforms the phase to γ phase of discrete nano-Al_2_O_3_. The adherent nano-Al_2_O_3_ particles remain as coated particles at 600 °C for 2 h and seem to move to the cathode lattice upon heat treatment such as at 600 °C for >4 h. This observation is consistent with the findings of Dogan, who observed the transformation from “surface coating” to “dopants” by annealing at high temperature in LiCoO_2_ but not in Li(Ni_0.__5_Co_0.2_Mn_0.__3_)O_2_ [30].

**Figure 5:**
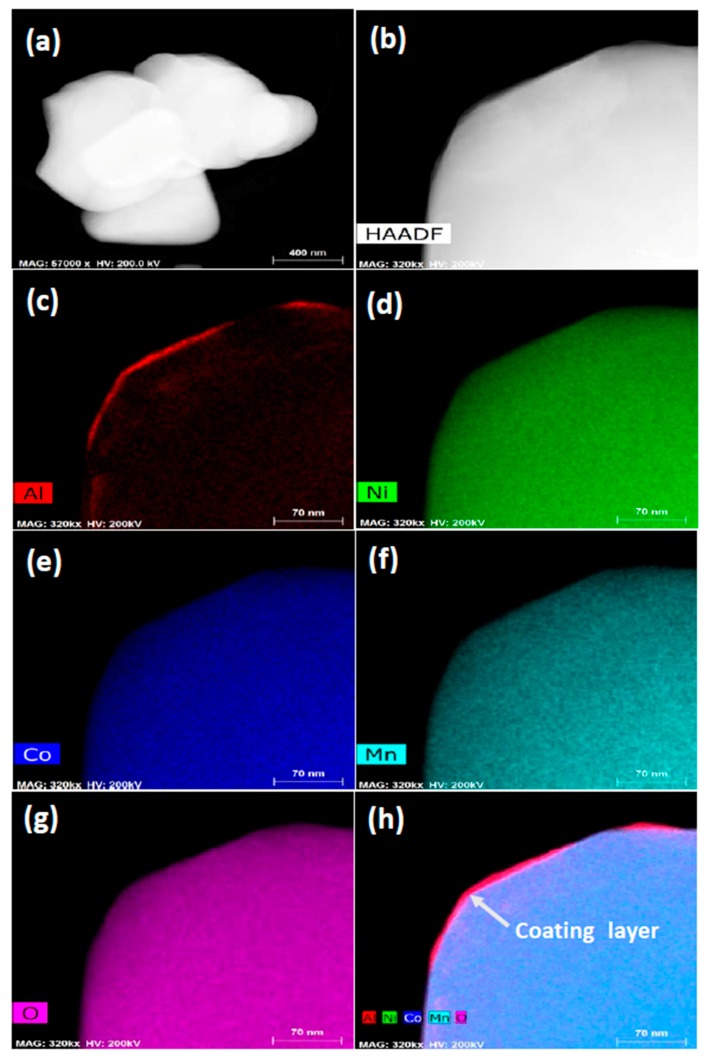
(**a**,**b**) Images of the EDS mapping region; and (**c**–**h**) the elemental mappings of the 0.08 wt % Al_2_O_3_-coated Li(Ni_0.6_Co_0.2_Mn_0.2_)O_2_ sample annealed at 600 °C for 4 h.

The elemental distribution of the Li(Ni_0.6_Co_0.2_Mn_0.2_)O_2_ samples in Figure 4 was studied using an EDS under STEM mode. As shown in Figure 5, Figure 5a is an image of the Li(Ni_0.6_Co_0.2_Mn_0.2_)O_2_ sample with the 0.08 wt % Al_2_O_3_ coating that had been annealed at 600 °C for 4 h, Figure 5b is a high-angle annular dark-field (HAADF) image of the EDS mapping region, and Figure 5c–h shows the distribution of Al, Co, Mn, Ni, and O in HAADF image, respectively. All elements are uniformly distributed on the surface of the Al_2_O_3_-coated Li(Ni_0.6_Co_0.2_Mn_0.2_)O_2_ particles. EDS mapping of Al shows a uniform distribution on the surface, except for bright bands at the edges as shown in Figure 5c and the Al_2_O_3_ coating layer (red color, 10–15 nm in thickness) was confirmed from Figure 5h.

### 3.2. Electrochemical Properties

In order to study the electrochemical performance of Li(Ni_0.6_Co_0.2_Mn_0.2_)O_2_ samples coated with different amounts of Al_2_O_3_, coin cells were operated at various C rates within the cutoff voltage of 3.0–4.5 V.

Figure 2 shows the electrochemical properties of the uncoated and Al_2_O_3_-coated samples annealed at 600 °C for 4 h between 3.0 and 4.5 V. Figure 2a shows the discharge capacity versus the total number of cycles according to the experimental procedure, as explained before. For detailed analysis, initial charge-discharge curves, rate capability, and cycle performance are displayed in Figure 2b–d, respectively. Figure 2b shows the initial charge-discharge curves of the samples cycled at a rate of 0.05 C. The initial discharge capacities for the samples uncoated and coated with 0.06, 0.08, and 1.2 wt % Al_2_O_3_ were 206.1, 200.6, 206.9, and 199.6 mAh/g, respectively. As shown in Figure 2a, the 0.08 wt % Al_2_O_3_-coated sample exhibits a good rate capability. Figure 2c shows the change in discharge capacities of the uncoated sample and the Al_2_O_3_-coated sample at various C rates. The discharge capacities of the uncoated sample were 206.1, 200.7, 193.1, 181.7, 169.8, and 155.8 mAh/g at 0.05 C, 0.1 C, 0.2 C, 0.5 C, 1 C, and 2 C, respectively. Under the same conditions, the discharge capacities of the 0.08 wt % Al_2_O_3_-coated sample were 206.9, 204.3, 199.9, 189.5, 177.7, and 166.3 mAh/g. With an increase in the C rate, i.e., the current density, the discharge capacity of the uncoated and Al_2_O_3_-coated sample both decrease, while the coated sample demonstrates a smaller discharge capacity decrease than the uncoated sample. The first discharge capacities were 206.1 and 206.9 mAh/g for the uncoated and coated samples, respectively; these samples had similar initial capacities. However, their discharge capacities after five cycles at a rate of 2 C were 155.8 and 166.3 mAh/g, respectively, demonstrating a notable difference in their values. The Al_2_O_3_-coated sample had a far greater improved rate capability than the uncoated sample. It is well known that the high rate capability of the coated sample is primarily due to the Al_2_O_3_ coating which protects the cathode material from reacting with electrolyte [24,31]. After being cycled from 0.05 C to 2 C, the recovered discharge capacity of 187.5 mAh/g at 0.5 C is almost identical to the initial discharge capacity at 0.5 C. This result indicates that there is good structural stability in the coated sample. The cycling performances of the uncoated and Al_2_O_3_-coated samples at 0.5 C over 3.0–4.5 V are shown in Figure 2a,d. The 0.08 wt % Al_2_O_3_-coated sample after 30 cycles also exhibited the best capacity retention of 94.5%, while the capacity retention of the uncoated sample was 90.8%. This indicates that the cycling stability of the 0.08 wt % Al_2_O_3_-coated sample could be improved by Al_2_O_3_ coating.

**Figure 6:**
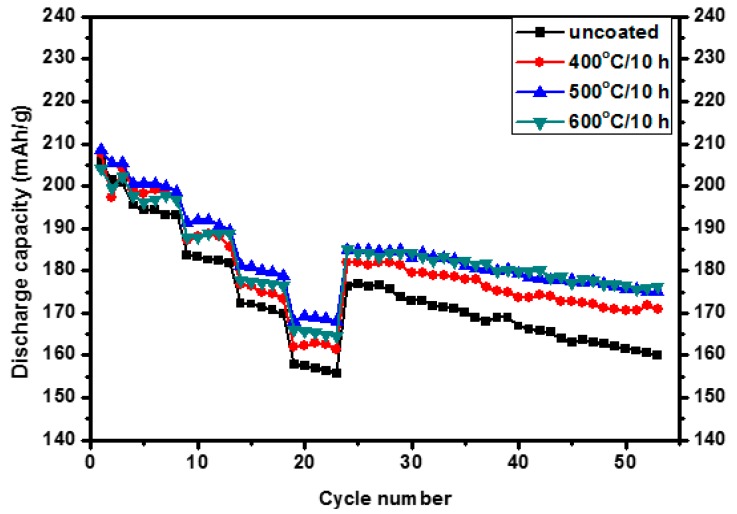
Electrochemical performance of the uncoated and 0.08 wt % Al_2_O_3_-coated Li(Ni_0.6_Co_0.2_Mn_0__.2_)O_2_ samples annealed at different temperatures for 10 h and cycled between 3.0 and 4.5 V at various C rates.

**Figure 7:**
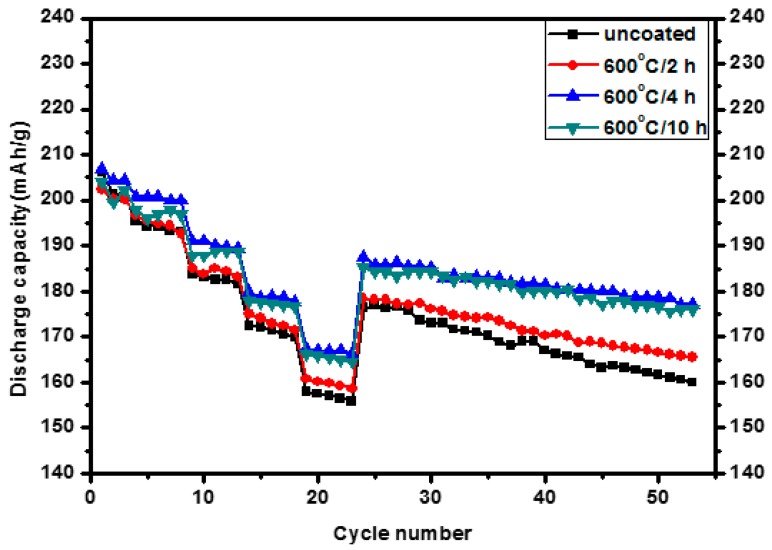
Electrochemical performance of the uncoated and 0.08 wt % Al_2_O_3_-coated Li(Ni_0.6_Co_0__.2_Mn_0.2_)O_2_ samples annealed at 600 °C for different holding times and cycled between 3.0 and 4.5 V at various C rates.

The effects of the temperature and holding time of heat treatment on the electrochemical properties of the Al_2_O_3_-coated samples are shown in Figure 6 and Figure 7, respectively. As shown in Figure 6, by increasing the temperature from 400 to 500, and 600 °C with the fixed duration of 10 h, the rate capability was improved for all the annealed samples, whereas the one at 500 °C showed better rate capability than that at 600 °C. But they became showing similarly improved cycling performances. Considering the SEM images in Figure 4, the coated nano-Al_2_O_3_ particles work better than the doped Al_2_O_3_ in the rate capability, but both improve the cycling performance similarly. As per holding time effects as shown in Figure 7, significant improvement in electrochemical performances were observed for both 4 h and 10 h at almost same extent, whereas only moderate improvement is observed for 2 h. Duration time of 4 h at 600 °C shows improvement as good as 10 h in both rate capability and cycling performance. 

## 4. Conclusions

In the present study, Li(Ni_0.6_Co_0.2_Mn_0.2_)O_2_ cathode materials were coated with a relatively small amount of Al_2_O_3_ by simple surface modification in water media. The uncoated and coated Li(Ni_0.6_Co_0.2_Mn_0.2_)O_2_ powders formed a polycrystalline hexagonal phase with a space group R$\bar{3}$m. From SEM observation, it was confirmed that the particles of Li(Ni_0.6_Co_0.2_Mn_0.2_)O_2_ cathode material were agglomerated and the discrete Al_2_O_3_ nano-particles of 10–15 nm were uniformly coated on the surface of the cathode material. The electrochemical properties of the samples were improved through Al_2_O_3_ coating of very low concentration; the optimum amount of Al_2_O_3_ additive was 0.08 wt % and the best heat treatment condition was 600 °C for 4 h. In the case of the 0.08 wt % Al_2_O_3_-coated Li(Ni_0.6_Co_0.2_Mn_0.2_)O_2_ sample annealed at 600 °C for 4 h, the initial discharge capacity at a rate of 0.05 C over 3.0–4.5 V was 206.9 mAh/g and the capacity retention at 0.5 C after 30 cycles was 94.5%. It could be concluded that the Li(Ni_0.6_Co_0.2_Mn_0.2_)O_2_ coated with nano-Al_2_O_3_ by simple surface modification and annealed at 600 °C for 4 h demonstrated improved electrochemical properties, including initial discharge capacity, rate capability, and cyclic performance. Finally, 0.08 wt % Al_2_O_3_-coated Li(Ni_0.6_Co_0.2_Mn_0.2_)O_2_ fabricated by the above method could be commercially used for the cathode material of lithium-ion secondary batteries.
